# The Experience of Mindfulness-Based Stress Reduction on Menopausal Symptoms, Sleep Disturbance, and Body Image among Patients with Breast Cancer—A Qualitative Study

**DOI:** 10.3390/curroncol30010097

**Published:** 2023-01-16

**Authors:** Yun-Chen Chang, Gen-Min Lin, Tzuhui Angie Tseng, Elsa Vitale, Ching-Hsu Yang, Ya-Ling Yang

**Affiliations:** 1 School of Nursing and Graduate Institute of Nursing, China Medical University, Taichung 40604, Taiwan; 2 Nursing Department, China Medical University Hospital, Taichung 404327, Taiwan; 3 Department of Medicine, Hualien-Armed Forces General Hospital, Hualien 97144, Taiwan; 4 Tri-Service General Hospital, National Defense Medical Center, Taipei 11490, Taiwan; 5 Department of Environmental and Cultural Resources, National Tsing Hua University, Hsinchu 30063, Taiwan; 6 Mental Health Center, ASL (Local Health Authority) Bari, 70026 Bari, Italy; 7 Department of Emergency Medicine, Hsinchu Mackay Memorial Hospital, Hsinchu 30071, Taiwan; 8 School of Nursing, College of Medicine, National Taiwan University, Taipei 10051, Taiwan

**Keywords:** breast cancer, mindfulness-based stress reduction (MBSR), menopausal, sleep disturbance, body image

## Abstract

Background and Aim: The objective was to decrease patient menopausal symptoms, sleep disturbance, and body image using a nonpharmacological therapy for cultivating key healthy lifestyle habits in patients with breast cancer. Materials and Methods: The participants were 26 women with breast cancer who had recently received structured mindfulness-based stress reduction (MBSR) training in a clinical trial. Focus groups and interviews were conducted, during which the participants were asked semistructured, open-ended questions regarding the experiences of MBSR. Results: The participants indicated that MBSR helped them to alleviate hot flashes and night sweats, and improve sleep quality and be more at ease with the external aspect of their body. On the other hand, during MBSR intervention in a group manner, the participants felt more psychological support and an outlet for sharing negative emotional experiences. Conclusion: This study identified the short-term benefits associated with group-based MBSR for women with breast cancer. In addition, our research identified the difficulties of intervention measures and coping methods. The study described the benefits of MBSR for patients with breast cancer. The findings of this study will help nursing staff identify the main coping menopausal symptoms and control negative mental health.

## 1. Introduction

Body image is generally defined as the subjective image of an individual’s own appearance established by noticing the reactions of others and self-observation [1,2]. Body image is a multi-dimensional structure, ranging from reconstructive surgery to eating disorders, and can occur in patients with breast cancer [2,3,4], and patients with breast cancer undergoing treatment with distorted body images may be profoundly affected in terms of their self-expression, including fear of thinking about the future, feelings of hopelessness, and disturbances in self-regulation [5,6]. Furthermore, cancer patients may experience domestic violence due to the contempt exhibited by their intimate partners [7]. However, negative body image and emotions can be improved through the use of complementary and alternative interventions [3].

The study showed that approximately 60% to 100% of breast cancer survivors experience at least one menopausal symptom [8]. Among menopausal symptom clusters, sleep disturbance was the biggest problem for patients with breast cancer, which was associated with increased risk of incident breast cancer [9], more physical pain, lower energy and vitality for performing daily activities, and poorer mental health [10]. A study reported that women with short sleep duration had higher risk of being diagnosed with breast cancer compared with women who slept at least 7 h per day [11]. Studies have also indicated that depression and sleep disorders can occur concurrently [12]. Although the aforementioned symptoms can be treated with specific medications, patients with breast cancer cannot use such medications because they may cause tumor recurrence, cardiovascular disease, dry mouth, sleep disturbance, and nausea [13,14]. Therefore, various forms of nonpharmacological behavioral health interventions have been studied, among which mindfulness-based stress reduction (MBSR) is widely used and has been proven to be safe, effective, and feasible. The MBSR program, developed by Dr. Kabat-Zinn, is based on mindfulness meditation that focuses on entering a state of open awareness as a form of moment-to-moment awareness and knowledge and on developing awareness of one’s own experience [15].

A qualitative study examined the benefits and barriers of MBSR from the perspective of patients with breast cancer; this international study reported that patients with breast cancer felt less fearful about cancer recurrence and identified with the suffering of others during the first two sessions of an MBSR program when they underwent the program with a supportive peer group [16]. Additionally, one study also suggested that practicing MBSR may be beneficial for maintaining mental health during unexpected adverse events, such as the COVID-19 lockdown [17]. However, in the context of MBSR, people in Asia seldom leverage the support of peer groups to share their personal perspective because of the conservative culture in Asia [6]. Therefore, appropriate guidance is required to help them share their personal experiences and express their feelings. Moreover, in addition to the narratives shared by participants, the observation of participants is a crucial method for collecting qualitative data. An advantage of focus groups is that such groups consist of acquaintances; thus, people feel at ease and can interact with each other in a normal manner.

A Canadian scholar once stated that social discomfort caused by body image problems may lead to social isolation, which leads to higher morbidity and mortality [18]. Having a good body image can promote better treatment and disease outcomes as well as a better ability to cope with a cancer diagnosis [18]. Our research is important because it helps to fill a gap in the current literature on positive body image in women with breast cancer [19]. Despite the increasing attention given to this topic in recent years, existing research on the subject has largely concentrated on therapeutic approaches [20], with a limited focus on the impact of mindfulness-based stress reduction (MBSR) interventions specifically. Our study, in contrast, aims to fill this gap by specifically exploring the effects of MBSR on sleep quality and emotional well-being, providing a more comprehensive understanding of this field. Our study illuminates the experiences and perspectives of women with breast cancer, providing valuable insights that can inform the development of clinical practices that aim to improve the quality of life for individuals affected by this disease. Through this research, we aim to contribute to the ongoing efforts to advance breast cancer care and enhance the outcomes for those impacted by this illness.

### Mindfulness Mechanisms

Baer (2003) argues that key mechanisms of mindfulness include exposure, cognitive change, self-management, relaxation, and acceptance [21]. In 2006, Shapiro et al. proposed that the mechanism of mindfulness encompasses three components, including intention, attention, and attitude, referred to as IAA [22]. The first core concept is intention: there is a possibility for change only if there is intention because intention can affect a person’s continuity. As long as one practices continuously, one can feel the change. The second core concept is attention: the core of mindfulness is attention, and attention itself has therapeutic effects. If the ability for self-regulation is sufficient, then there can be positive reinforcement effects between IAA. The third core concept is attitude: assuming that one’s internal and external experiences are learnable and do not require evaluation or explanation, even if the situation happens to be contrary to one’s wishes or expectations, one’s attitude can still be accepting and maintain a friendly and open attitude. These three mindfulness concepts complement each other in a process of mutual interaction, forming a holistic concept that operates in a cyclical and intertwined manner moment to moment. Emphasis is placed on the ability to focus internally and closely pay attention to the present moment and the sensations and thoughts of the body and mind without subjectively commenting on them. This allows for the maintenance of mindfulness, which promotes balance and well-being by reducing stress [22]. Mindfulness operation leads to a change in “reperceiving”, which means that the individual’s original subjectivity of perception transforms into an objectivity. It allows one to step out of one’s consciousness and observe one’s experiences more clearly and objectively. Additionally, by going through the process of reperceiving, it increases self-regulation and cognitive recognition of what really matters, and it improves cognitive, emotional, and behavioral adaptability and acceptance of both positive and negative thoughts and ultimately enhances positive physical and mental well-being [22].

## 2. Materials and Methods

### 2.1. Study Design

We conducted semi-structured interviews with breast cancer patients. In the present qualitative study, constructivist grounded theoretical methods [23] were used to examine an MBSR program for women undergoing breast cancer treatment.

### 2.2. Sampling and Eligibility Criteria

We used theoretical sampling [24] to build a theory or understand the nature of problems that emerge. The present study explored the first-hand experiences of patients with breast cancer. Recruitment was conducted by placing advertisements on the website of the breast cancer peer support group and breast surgery clinic of the study hospital. Patients were recruited if they had received a diagnosis of breast cancer (stages 0–IV) within the previous 5 years and were aged 18 years or older. Patients who were diagnosed with psychosis and were taking medication for this illness were excluded.

### 2.3. Procedure

The present study was approved by an ethics committee. The participants provided written informed consent, and nutritional supplements were provided as an incentive prior to implementation of the MBSR program.

Researchers applying grounded theory must choose a field of interest and a problem to solve, and no solution is absolutely correct or incorrect [25]. Therefore, during the interviews conducted in the present study, the focus was on obtaining the responses of patients by asking them the following nondirective, open-ended questions related to their experiences with the MBSR program:

1. During the MBSR intervention, what have been the most notable changes that you have experienced with respect to aspects such as menopausal symptoms and body image?
2. What difficulties did you encounter during the program and how did you solve them? When the interviewers determined that an interview could not generate new themes (i.e., saturation had been achieved), data collection was stopped.

#### 2.3.1. Participation in Focus Groups

A total of 32 participants met the inclusion criteria for present study, 3 of whom lived too far away and 3 of whom had family factors, so a total of 26 participants were interviewed. Participants were divided into three focus groups (Group A, *n* = 5; Group B, *n* = 6; and Group C, *n* = 7). To acquire a representative sample of personal opinions and intervention experiences, eight participants were asked to participate in in-depth interviews instead of a focus group. Patients who consented to participate in the focus groups met in a well-lit and clean yoga classroom following completion of the MBSR program. Of these patients, 69.2% held a college degree or higher level of education, the majority (61.5%) had been diagnosed with invasive breast cancer at Stage II, and all had completed adjuvant therapy, including surgery, chemotherapy, and radiotherapy. Some of these patients were still undergoing hormone treatment at the time of the focus groups. Because of the camaraderie among the participants, they were willing to discuss and share their experiences in public. Notes and video recordings of the focus group discussions were made. The video recordings were transcribed verbatim for analysis and reviewed carefully. To protect the privacy of all subjects, the recordings were transcribed using pseudonyms.

#### 2.3.2. MBSR Intervention

The original 8-week MBSR program was developed by Kabat-Zinn [15]. For the participants’ convenience, our MBSR program was conducted over 6 consecutive weeks and consisted of 2 h sessions and home-based practice; meditation instruction was provided in MP3 and YouTube format, and meditation sessions lasting at least 10–15 min were conducted five or six times per week. The participants were required to write down their personal experiences on a sheet of paper. Intensive MBSR training was administered by a qualified instructor trained in the professional MBSR system and a research assistant who recorded patients’ feelings about specific topics, including their verbal and nonverbal responses.

### 2.4. Data Analysis

Data saturation was achieved with three focus groups and eight interviews. To identify and organize emerging themes, constructivist ground theory was similar to Glaser and Strauss’s (1976) developed ground theory [24] in data analysis; that is, data collection and analysis took place concurrently with each informing the other. Once the analysis of the participants was completed, the researchers set aside any assumptions or personal biases. Interview documents were imported into NVivo v12 (QSR International), a software program that facilitates the management and analysis of qualitative data. Data collection and analysis were performed in a periodic, interrelated manner throughout the study process. The focus group and interview data collected from the participants were compared line by line to identify recurring patterns in the data and coded independently by three researchers. Using a constant comparison method, we systematically compared and coded for similarities and differences within and between transcripts, starting this rolling comparison process from the start of data collection. The interview guide has been edited to explore new themes emerging from the data. The coded data was put together to form a collective image of the responses in all interview data, and the process generated themes such that a theoretical framework could be established using the data. To achieve our goals, the final narrative was returned to the participants, and further analysis of the data was added so that they could confirm that the interpretation of the interviews and observations was correct [26].

### 2.5. Rigor

The intervention was instructed by a qualified clinical psychologist with specialized training in psychology at the Departmental Cancer Center, Medical Center in Taiwan. To maintain the credibility and trustworthiness of the mindfulness program, reflexivity remained during the data collection and analysis [24]. Field recordings were used to capture nuances in participants’ nonverbal responses and to enhance the authenticity of verbal citations. The instructor evaluated the transfer of MBSR skills by discussing the material with the patients, who recorded the duration of their own self-reflection practice weekly in order to maintain accuracy in reporting the participants’ emic (insider) perspective throughout the data collection.

## 3. Results

The sample comprised 26 female patients with breast cancer. Participant characteristics are described in Table 1. They had an average age of 45.65 (standard deviation [SD] = 8.09) years. The average time between their diagnosis of cancer and the present study was 26.35 (SD = 19.62) months. Most had a college education (17, 65.4%), followed by a junior college education (5, 19.2%); 61.5% were diagnosed with stage II breast cancer. Those receiving or who had had received chemotherapy or hormonal therapy accounted for 34.6% and 50%, respectively. A total of 12 (46.2%) patients answered yes for the option regarding sleeping pills, and 14 (53.8%) answered no.

After data collection and analysis, the code was divided into higher-level concepts and then categorized. These categories became the basis of a theoretical model. The final grounded theory is presented in Table 2; it describes the main themes relating to the symptoms caused by treatment, initial behavioral pattern of mindfulness, and the experience of MBSR among patients with breast cancer. Six subthemes were identified, namely (a) sleep disturbance following diagnosis or treatment, (b) negative body image and sexual problems among patients with breast cancer, (c) self-awareness of the stress of life but still trying to practice, (d) the support of a patient peer group, (e) discovery of the benefits of implementing mindfulness in daily life, (f) additional benefits of the mindfulness intervention.

### 3.1. Symptoms Caused by Treatment

#### 3.1.1. Sleep Disturbance following Diagnosis or Treatment

The surgery performed on patients with breast cancer can damage their lymph nodes or blood vessels, preventing the circulation of lymph fluid within the body and causing the accumulation of fluid at the extremities. Psychological or functional discomfort may occur throughout the course of lymphedema [27]. Out of the participants we included, approximately 19.2% had experienced damage to their lymph nodes or blood vessels. One patient reported the following:

“My arms are often swollen like an elephant’s legs (the patient rolls up her sleeves to show us her arms), and my limbs feel different depending on the weather.”

(Adelina)

Hormonal therapy can cause side effects such as menopausal symptoms, including hot flashes, night sweats, and frequent urination. Specifically, 25% of the patients reported that their sleep quality worsened after undergoing hormonal therapy. Three patients said the following:

“I have experienced sleep disturbance, and I can’t fall asleep even after taking sleeping pills. I wake up early in the morning and feel considerable pain [the patient frowns and clenches her fists.”

(Jessica)

“The side effect of taking Tamoxifen (Nolvadex) is that I’m always going to the bathroom at midnight and sometimes three or four times a night. This affects my sleep quality substantially.”

(Hellen)

“Taking Tamoxifen (Nolvadex) causes me to wake up two or three times around midnight because of hot flushes and frequent urination; thus, I feel exhausted the next day.”

(Emma)

#### 3.1.2. Negative Body Image and Sexual Problem among Patients with Breast Cancer

Surgical removal of the breast often affects a woman’s body image and sexuality, and the side effects of chemotherapy could lead to negative physical and mental reactions and indirectly affect sexual problems [6,28]. Hormone therapy could reduce sexual desire, orgasm, and genital intercourse frequency in breast cancer survivors. The international literature showed that 61% of women with breast cancer and their husbands become estranged after treatment [29]. However, only two participants in our study reported experiencing the above problem. Possible reasons for this could include the fact that East Asian women tend to be relatively conservative when it comes to discussing sexual issues, as well as the fact that the interview mode used was a focus group format, which may have made participants feel uncomfortable discussing this issue in front of others [6]. As one woman stated:

“The scar on my body is like a centipede, I was afraid of seeing the mirror, so I removed all the mirrors in the house…I don’t let my husband touch my body because I’m worried he doesn’t love me anymore.”

(Jane)

### 3.2. Initial Behavioral Pattern of Mindfulness

#### 3.2.1. Self-Aware of the Stress of Life but Still Trying to Practice

A beginner practitioner of mindfulness requires a degree of persistence and confidence to adapt to mindfulness practice. Several patients said that they were always so busy and preoccupied with daily matters that they forgot to practice mindfulness. Nevertheless, they were still willing to spend a few minutes each day to practice mindfulness at their own pace. Three patients said the following:

“I forgot to do mindful breathing at the beginning…I thought a lot at the beginning and would zone out during the process, but I was being able to absorb the lessons later. Mindful breathing helps to gradually slow my respiratory rate.”

(Lucy)

“I currently have to work and undergo regular targeted therapy, so I don’t have much time to practice, but I will try mindful breathing and body scanning when I have the time. In the beginning, I couldn’t sense my body, but through rehabilitation…”

(Fiona)

“I don’t practice often because I’m too busy…I either have work to do or just want to sleep when I have time to spare. It is quite difficult for me to practice mindfulness in my daily life, but I can take time to practice it for a few minutes each day.”

(Aileen)

#### 3.2.2. The Support of a Patient Peer Group

In general, it is possible that some participants may have found the mindfulness techniques more beneficial than others or that they may have had different levels of engagement or adherence to the intervention. Some participants may have had a better response to certain techniques, such as meditation or yoga, while others may have found other techniques, such as body scan or mindful eating, to be more effective. Additionally, participants may have varied in their overall well-being, general mental health, coping abilities, or even the stage of cancer, which could affect the level of benefit they experienced. During the MBSR program, the patients who learned about the experiences of other patients could have developed various thoughts and attitudes about life. In addition to the stress of undergoing breast cancer treatment, patients with children experienced more stress in their lives [30]. One patient said the following:

“Because of cancer, it is difficult for me to find a job…Some people have experienced severe side effects after chemotherapy and radiotherapy, including dizziness, soaring blood pressure, and insomnia. I have to help myself by setting goals while confronting the hardships in life…The goals I set are to live for more than 5 years and to train my son to take care of himself and live independently after I’m gone.”

(Cherry)

The patients must be able to detect and control their emotions because they affect the people around them. In this regard, the patients said that the patient support groups provided them with psychological support and an outlet for sharing their experiences. Three patients said the following:

“If you don’t control your temper well after you get sick, the people around you will be affected. I hope that this group can continue in the future so that everyone can share their experiences at any time.”

(Kelly)

“We can all help ourselves. Mindfulness courses help us to gradually understand that we must rely on ourselves to solve problems and must focus on the present. All difficulties can be overcome over time. Health is the most important thing.”

(Rebecca)

“Actually, I was quite happy when I heard that I was sick. This was because I was so overwhelmed by life that becoming sick allowed me to take a rest. I deal with my difficulties and anxiety by accepting them, focusing on the present, and getting support by interacting with other patients. There are always trials to overcome in our lives for various reasons. They force us to grow and bring fulfillment in our lives.”

(Danielle)

### 3.3. The Experience of MBSR among Patients with Breast Cancer

#### 3.3.1. Discovery of the Benefits of Implementing Mindfulness in Daily Life

Most patients with breast cancer live a fast-paced life. After they had attended a series of mindfulness sessions, 76.9% of the participants who were patients with breast cancer living a fast-paced lifestyle reported that the mindfulness intervention was beneficial to them. Three patients said the following:

“I am impressed by mindful eating, meditation, stretching, and walking because all these activities remind me that I need to slow down; in particular, when I’m tasting raisins, I find that the practice of mindfulness is very good but difficult to implement because I’m always in a rush to finish my meal.”

(Chloe)

“When I touched the wound of my mastectomy, I was very low self-esteem. I wanted to cry every night (eyes were red), but through breathing adjustment and body scans, it seemed that the sadness gradually diminished.”

(Abby)

“I used to hate having sex with my husband, but through “mindfulness” sex, I felt involved in the whole sex process…”

(Una)

During the process of writing a diary, the patients focused on describing the topics that they really cared about, mastering their thoughts, figuring out causes and effects, and developing new ideas for solving problems. For the patients, their diaries also served as an external portable storage device in which they could store their emotions and thoughts. During the discussion, several patients said that writing in a diary was an emotional outlet for them. One patient said the following:

“In mindfulness practice, I can relax…Everything happens at a slow pace. I set goals for myself, keep records, and write in a diary every day. Doing only one thing at a time is less distracting for me.”

(Wendy)

Most of the participants were beginner practitioners of MBSR. They knew nothing about MBSR prior to taking the course. Several participants thought it was about positive thinking, whereas others thought it was an effective yoga course. However, after 6 weeks, one of the patients discovered that numerous aspects of the MBSR program had helped her detect positive feelings relating to her body and emotions. She said the following:

“I’m very impressed with the mindfulness program because I initially thought that this course was just a yoga course; however, I gained a lot by learning various mindfulness skills after joining the course.”

(Zera)

#### 3.3.2. Additional Benefits of the Mindfulness Intervention

Approximately 90% of patients frequently have complex thoughts. Participants chose mindfulness techniques that suited them. Core mindfulness techniques such as meditation and body scans were the most popular, with a preference rate of 80.8% and 73.1% respectively. Other participants preferred techniques such as yoga and mindful eating, which have positive effects on the psychological well-being of breast cancer patients. Additionally, mindfulness practice is not limited by location and can be practiced at home or in other places such as hospitals. Two attendees stated the following:

“Sometimes I wake up halfway through my sleep at night because I always have so many thoughts that I can’t let go of. Through mindfulness, I can relax more…Thus, I want to tell everyone to let go of their obsessions. This is more beneficial for us.”

(Vicky)

“During my hospitalization, I often felt very irritable, but after I performed mindfulness body scans and adjusted my breathing before going to bed, I found that the distracting thoughts in my mind slowly subsided.”

(Tracy)

## 4. Discussion

The present study aimed to explore whether MBSR can improve the menopause symptoms, sleep quality, body image, and perception of mindfulness training in patients with breast cancer. At present, few studies have explored whether mindfulness can improve the body image or negative appreciation of body scar of patients with breast cancer. Only one similar study examines psychoeducation on premenopausal women with excess body weight, and the results found a decreased negative evaluation of the body [31]. On the other hand, there are also patients with breast cancer who said that it is also helpful to improve sexual relationships. The findings of current study were consistent with the results of previous study [32], which indicated that MBSR could improve female sexual function, such as arousal, lubrication, and satisfaction [32].

The patients with breast cancer enrolled in the present study were considerably young; their average age was 45.65 years. They had to work hard to overcome stress pertaining to their family, work, and treatment. Therefore, approximately 2 participants reported frequently forgetting to practice mindfulness techniques. As a result, they would make up for it by practicing the techniques the following day. Several participants said that even when they were resting, their brains were still operating as if they were working. This phenomenon affected their perception of their body and breathing during the practice of mindfulness. However, the aforementioned phenomenon is a normal one. The founder of MBSR, Kabat-Zinn, stressed that the main concept of MBSR is not for the person practicing it to criticize any feelings or thoughts but to accept what they are observing [15]. When people become more proficient at mindfulness, they can benefit from physical and mental stability and an improvement in their quality of life in the long term [33].

Numerous patients said that they required group support through which they could share their experiences, exchange information, and acquire emotional support [16]. This finding of the present study aligns with that of previous qualitative research in indicating that support groups play a key role in the process of MBSR learning. MBSR appears to create an atmosphere in which participants can receive social support within a group.

During the first MBSR session, the participants were asked to eat raisins, and they reported that this activity was particularly difficult to perform but provided a positive experience. Mindful eating can boost the parasympathetic nervous system and promote homeostasis in the autonomic nervous system, which is essential for optimal digestive function. The helpful mindfulness skills that the participants had learned can serve as a reference for patients and clinical medical staff who intend to launch MBSR programs in the future.

Following breast cancer treatment, capillary permeability can be increased due to reduced clearance of inflammatory mediators that promote low-grade inflammation; consequently, complications linked to breast-cancer-related lymphedema may occur [34]. Numerous factors influence the occurrence of insomnia among women with breast cancer; they include endocrine therapy, hot flashes, cancer-related pain, and fear of recurrence [35]. The focus groups revealed that the MBSR intervention had a positive effect on the sleep quality of the participants; this finding echoes that of a systematic review and meta-analysis, which produced moderate evidence that mindfulness interventions have a positive effect on sleep quality [36].

In the present study, a patient who had brain and bone metastases said that she often had a fear of death but could gradually calm herself down by practicing mindfulness. The third main theme of the present study was the perception benefits of implementing mindfulness, which is an MBSR mechanism proposed by Hölzel [37]. Specifically, he proposed that mindfulness training leads to alteration of the frontolimbic network in the brain, which is essential for emotional regulation [38]. Notably, in a longitudinal survey conducted by Madson et al. (2018), the MBSR program that was conducted improved the mental health and feeling of well-being of participating patients, with these feelings found to last for up to 2 years [39].

### 4.1. Limitations

The present study has several limitations that must be acknowledged. One limitation is that qualitative data analysis can be subjective. While themes were chosen based on scientific analysis, they were still somewhat affected by the researchers’ involvement. To produce more objective and complete results, future research should include objective physical tests as indicators. Another limitation is the absence of control groups and quantitative measurements, which raises concerns about the generalizability of the study’s subjective reports. Additionally, the small sample size also raises questions about the generalizability of the findings to a larger population.

### 4.2. Implications for Nursing Practice

Excellent physical health, mental health, and quality of life require excellent sleep quality [40]. Studies have revealed that the experience of receiving a diagnosis of and being treated for breast cancer can cause considerable distress [41]; notably, the aforementioned experience can cause a high proportion of patients with breast cancer to experience one or more types of sleep deprivation [42]. To the best of our knowledge, patients with noninvasive breast cancer who experience sleep deprivation are more likely to develop invasive breast cancer; thus, nursing staff should strive to alleviate the sleep problems of such patients [43]. The present study can serve as a reference for nursing care providers with respect to continual assessment of menopausal symptoms and body image for patients receiving MBSR education. Furthermore, our results indicated that a group-based MBSR program can improve the emotional state of patients through implementation of a patient support group.

## 5. Conclusions

Our study aimed to understand the benefits of mindfulness-based stress reduction (MBSR) from the point of view of patients with breast cancer, how it affected their mental and physical distress, and how they perceived the skills they acquired from MBSR. Through this research, we sought to offer a holistic, non-pharmacological approach to improving the well-being of breast cancer patients. The findings of our study can be used by healthcare professionals and researchers to understand the potential benefits of MBSR for breast cancer patients, and to implement similar interventions in their own practices or studies. Furthermore, patients with breast cancer can gain insight about how MBSR skills can help them to cope with the challenges of their condition, especially in distress and mental well-being.

## Figures and Tables

**Table 1:** The clinical characteristics of patients (*N* = 26).

Characteristic	*N* = 26
Age (years), mean (SD)	45.65 (8.09)
Time since diagnosis (months), mean (SD)	26.35 (19.62)
Educational level, *n* (%)	
Senior high school	3 (11.5)
Junior college	5 (19.2)
College	17 (65.4)
Graduate institute	1 (3.8)
Cancer staging, *n* (%)	
Stage 0	1 (3.8)
Stage I	3 (11.5)
Stage II	16 (61.5)
Stage III	1 (3.8)
Stage IV	5 (19.2)
Treatment, *n* (%)	
Chemotherapy	9 (34.6)
Radiotherapy	1 (3.8)
Targeted therapy	2 (7.7)
Hormone therapy	13 (50)
Other	1 (3.8)
Use of sleeping pills, *n* (%)	
No	14 (53.8)
Yes	12 (46.2)

Abbreviations: SD, standard deviation.

**Table 2:** Themes and subthemes.

Themes	Subthemes
Symptoms caused by treatment	Sleep disturbance following diagnosis or treatment
	Negative body image and sexual problems among patients with breast cancer
Initial behavioral pattern of mindfulness	Self-awareness of the stress of life but still trying to practice
	The support of a patient peer group
The experience of MBSR among patients with breast cancer	Discovery of the benefits of implementing mindfulness in daily life
	Additional benefits of the mindfulness intervention

## Data Availability

The data presented are available on request from the corresponding author.

## References

[1] Dahl C.A.F., Reinertsen K.V., Nesvold I.L., Fosså S.D., Dahl A.A. (2010). A study of body image in long-term breast cancer survivors. Cancer.

[2] Thompson J.K., Heinberg L.J., Altabe M., Tantleff-Dunn S. (1999). Exacting Beauty: Theory, Assessment, and Treatment of Body Image Disturbance.

[3] Lewis-Smith H., Diedrichs P.C., Harcourt D. (2018). A pilot study of a body image intervention for breast cancer survivors. Body Image.

[4] Rezaei M., Elyasi F., Janbabai G., Moosazadeh M., Hamzehgardeshi Z. (2016). Factors Influencing Body Image in Women with Breast Cancer: A Comprehensive Literature Review. Iran. Red Crescent Med. J..

[5] Sebri V., Triberti S., Pravettoni G. (2020). Injured Self: Autobiographical Memory, Self-Concept, and Mental Health Risk in Breast Cancer Survivors. Front Psychol..

[6] Chang Y.C., Hu W.Y., Chang Y.M., Chiu S.C. (2019). Changes in sexual life experienced by women in Taiwan after receiving treatment for breast cancer. Int. J. Qual. Stud. Health Well-Being.

[7] Leite F.M.C., Oliveira A.G., Barbosa B.L.F.d.A., Ambrosim M.Z., Vasconcellos N.A.V., Maciel P.M.A., Amorim M.H.C., Furieri L.B., Lopes-Júnior L.C. (2022). Intimate Partner Violence against Mastectomized Women: Victims&rsquo; Experiences. Current Oncol..

[8] Mazor M., Lee K., Dhruva A., Cataldo J.K., Paul S.M., Melisko M., Smoot B.J., Levine J.D., Elboim C., Conley Y.P. (2018). Menopausal-Related Symptoms in Women One Year after Breast Cancer Surgery. J. Pain Symptom Manag..

[9] Cao J., Eshak E.S., Liu K., Muraki I., Cui R., Iso H., Tamakoshi A. (2019). Sleep duration and risk of breast cancer: The JACC Study. Breast Cancer Res. Treat..

[10] Zhang X., Sun D., Wang Z., Qin N. (2022). Triggers and Coping Strategies for Fear of Cancer Recurrence in Cancer Survivors: A Qualitative Study. Curr. Oncol..

[11] Lehrer S., Green S., Ramanathan L., Rosenzweig K.E. (2013). Insufficient sleep associated with increased breast cancer mortality. Sleep Med..

[12] Riemann D., Krone L.B., Wulff K., Nissen C. (2020). Sleep, insomnia, and depression. Neuropsychopharmacology.

[13] Breyer J.Z., Wendland E.M., Kops N.L., Caleffi M., Hammes L.S. (2018). Assessment of potential risk factors for breast cancer in a population in Southern Brazil. Breast Cancer Res. Treat..

[14] Rada G., Capurro D., Pantoja T., Corbalán J., Moreno G., Letelier L.M., Vera C. (2010). Non-hormonal interventions for hot flushes in women with a history of breast cancer. Cochrane Database Syst. Rev..

[15] Kabat-Zinn J. (1990). Full Catastrophe Living: The Program of the Stress Reduction Clinic at the University of Massachusetts Medical Center.

[16] Schellekens M.P., Jansen E.T., Willemse H.H., van Laarhoven H.W., Prins J.B., Speckens A.E. (2016). A qualitative study on mindfulness-based stress reduction for breast cancer patients: How women experience participating with fellow patients. Support Care Cancer.

[17] Accoto A., Chiarella S.G., Raffone A., Montano A., de Marco A., Mainiero F., Rubbino R., Valzania A., Conversi D. (2021). Beneficial Effects of Mindfulness-Based Stress Reduction Training on the Well-Being of a Female Sample during the First Total Lockdown Due to COVID-19 Pandemic in Italy. Int. J. Environ. Res. Public Health.

[18] Albert J.G., Lo C., Rosberger Z., Frenkiel S., Hier M., Zeitouni A., Kost K., Mlynarek A., Black M., MacDonald C. (2022). Biopsychosocial Markers of Body Image Concerns in Patients with Head and Neck Cancer: A Prospective Longitudinal Study. Curr. Oncol..

[19] Thornton M., Lewis-Smith H. (2021). “I listen to my body now”: A qualitative exploration of positive body image in breast cancer survivors. Psychol. Health.

[20] Guedes T.S.R., Dantas de Oliveira N.P., Holanda A.M., Reis M.A., Silva C.P., Rocha e Silva B.L., Cancela M.C., de Souza D.L.B. (2018). Body Image of Women Submitted to Breast Cancer Treatment. Asian Pac J. Cancer Prev..

[21] Baer R.A. (2003). Mindfulness Training as a Clinical Intervention: A Conceptual and Empirical Review. Clin. Psychol. Sci. Pract..

[22] Shapiro S.L., Carlson L.E., Astin J.A., Freedman B. (2006). Mechanisms of mindfulness. J. Clin. Psychol..

[23] Charmaz K. (2006). Constructing Grounded Theory: A Practical Guide through Qualitative Analysis.

[24] Glaser B.G., Strauss A.L., Strutzel E. (1968). The discovery of grounded theory; strategies for qualitative research. Nurs. Res..

[25] Glaser B.G. (1992). Basics of Grounded Theory Analysis: Emergence vs Forcing.

[26] Speziale H.S., Streubert H.J., Carpenter D.R. (2011). Qualitative Research in Nursing: Advancing the Humanistic Imperative.

[27] Vignes S. (2017). Lymphedema: From diagnosis to treatment. Rev. Med. Interne.

[28] Chang Y.C., Chang S.-R., Chiu S.-C. (2019). Sexual Problems of Patients With Breast Cancer After Treatment: A Systematic Review. Cancer Nurs..

[29] Brédart A., Dolbeault S., Savignoni A., Besancenet C., This P., Giami A., Michaels S., Flahault C., Falcou M.C., Asselain B. (2011). Prevalence and associated factors of sexual problems after early-stage breast cancer treatment: Results of a French exploratory survey. Psychooncology.

[30] Nomaguchi K.M., Milkie M.A. (2003). Costs and rewards of children: The effects of becoming a parent on adults’ lives. J. Marriage Fam..

[31] Czepczor-Bernat K., Brytek-Matera A., Staniszewska A. (2021). The effect of a web-based psychoeducation on emotional functioning, eating behaviors, and body image among premenopausal women with excess body weight. Arch. Womens Ment. Health.

[32] Chang Y.C., Lin G.M., Yeh T.L., Chang Y.M., Yang C.H., Lo C., Yeh C.Y., Hu W.Y. (2022). Impact of mindfulness-based stress reduction on female sexual function and mental health in patients with breast cancer. Support. Care Cancer.

[33] Banth S., Ardebil M.D. (2015). Effectiveness of mindfulness meditation on pain and quality of life of patients with chronic low back pain. Int. J. Yoga.

[34] Jensen M.R., Simonsen L., Karlsmark T., Bülow J. (2013). Microvascular filtration is increased in the forearms of patients with breast cancer-related lymphedema. J. Appl. Physiol..

[35] Kwak A., Jacobs J., Haggett D., Jimenez R., Peppercorn J. (2020). Evaluation and management of insomnia in women with breast cancer. Breast Cancer Res. Treat..

[36] Rusch H.L., Rosario M., Levison L.M., Olivera A., Livingston W.S., Wu T., Gill J.M. (2019). The effect of mindfulness meditation on sleep quality: A systematic review and meta-analysis of randomized controlled trials. Ann. N. Y. Acad. Sci..

[37] Holzel B.K., Carmody J., Vangel M., Congleton C., Yerramsetti S.M., Gard T., Lazar S.W. (2011). Mindfulness practice leads to increases in regional brain gray matter density. Psychiatry Res..

[38] Holzel B.K., Hoge E.A., Greve D.N., Gard T., Creswell J.D., Brown K.W., Barrett L.F., Schwartz C., Vaitl D., Lazar S.W. (2013). Neural mechanisms of symptom improvements in generalized anxiety disorder following mindfulness training. Neuroimage Clin..

[39] Madson L., Klug B., Madson L., Stimatze T., Eness-Potter K., MacDonald J. (2018). Effectiveness of mindfulness-based stress reduction in a community sample over 2 years. Ann. Clin. Psychiatry.

[40] Chattu V.K., Manzar M.D., Kumary S., Burman D., Spence D.W., Pandi-Perumal S.R. (2018). The Global Problem of Insufficient Sleep and Its Serious Public Health Implications. Healthcare.

[41] Campbell-Enns H., Woodgate R. (2015). The psychosocial experiences of women with breast cancer across the lifespan: A systematic review protocol. JBI Database Syst. Rev. Implement Rep..

[42] Koopman C., Nouriani B., Erickson V., Anupindi R., Butler L.D., Bachmann M.H., Sephton S.E., Spiegel D. (2002). Sleep disturbances in women with metastatic breast cancer. Breast J..

[43] Touitou Y., Reinberg A., Touitou D. (2017). Association between light at night, melatonin secretion, sleep deprivation, and the internal clock: Health impacts and mechanisms of circadian disruption. Life Sci..

